# Berberine Sulfate Attenuates Osteoclast Differentiation through RANKL Induced NF-κB and NFAT Pathways

**DOI:** 10.3390/ijms161125998

**Published:** 2015-11-13

**Authors:** Lin Zhou, Fangming Song, Qian Liu, Mingli Yang, Jinmin Zhao, Renxiang Tan, Jun Xu, Ge Zhang, Julian M. W. Quinn, Jennifer Tickner, Jiake Xu

**Affiliations:** 1 School of Pathology and Laboratory Medicine, the University of Western Australia, Perth, WA 6009, Australia; 20463692@student.uwa.edu.au (L.Z.); songfangming@gxmu.edu.cn (F.S.); liuqian@gxmu.edu.cn (Q.L.); 21159333@student.uwa.edu.au (M.Y.); jennifer.tickner@uwa.edu.au (J.T.); 2 Research Centre for Regenerative Medicine, Guangxi Key Laboratory of Regenerative Medicine, Guangxi Medical University, Nanning 530021, China; zhaojinmin@gxmu.edu.cn; 3 Pharmaceutical college, Guangxi Medical University, Nanning 530021, China; 4 Department of Orthopaedic Surgery, the First Affiliated Hospital of Guangxi Medical University, Nanning 530021, China; 5 Institute of Functional Biomolecules, Medical School, Nanjing University, Nanjing 210093, China; rxtan@nju.edu.cn; 6 Research Center for Drug Discovery (RCDD), School of Pharmaceutical Sciences, Sun Yat-Sen University, 132 East Circle at University City, Guangzhou 510006, China; junxu@biochemomes.com; 7 Institute for Advancing Translational Medicine in Bone & Joint Diseases, School of Chinese Medicine, Hong Kong Baptist University, Kowloon Tong, Hong Kong; zhangge@hkbu.edu.hk; 8 The Garvan Institute of Medical Research, Darlinghurst, NSW 2010, Australia; j.quinn@garvan.org.au

**Keywords:** berberine sulfate, osteoclast, RANKL, NF-κB and NFAT pathway

## Abstract

Osteoporosis, a metabolic bone disease, is characterized by an excessive formation and activation of osteoclasts. Anti-catabolic treatment using natural compounds has been proposed as a potential therapeutic strategy against the osteoclast related osteolytic diseases. In this study, the activity of berberine sulfate (an orally available form of berberine) on osteoclast differentiation and its underlying molecular mechanisms of action were investigated. Using bone marrow macrophages (BMMs) derived osteoclast culture system, we showed that berberine sulfate at the dose of 0.25, 0.5 and 1 μM significantly inhibited the formation of osteoclasts. Notably, berberine sulfate at these doses did not affect the BMM viability. In addition, we observed that berberine sulfate inhibited the expression of osteoclast marker genes, including cathepsin K (Ctsk), nuclear factor of activated T cells cytoplasmic 1 (NFATc1), tartrate resistant acid phosphatase (TRAcP, Acp5) and Vacuolar-type H+-ATPase V0 subunit D2 (V-ATPase d2). Luciferase reporter gene assay and Western blot analysis further revealed that berberine sulfate inhibits receptor for activation of nuclear factor ligand (RANKL)-induced NF-κB and NFAT activity. Taken together, our results suggest that berberine sulfate is a natural compound potentially useful for the treatment of osteoporosis.

## 1. Introduction

Bone remodelling is a continuous and dynamic process regulated by osteoclastic bone resorption and osteoblastic bone formation [1]. Overproduction and/or excessive activation of osteoclasts can lead to osteolytic bone diseases, such as postmenopausal osteoporosis and Paget’s disease of the bones [2].

Osteoclasts are multinuclear giant cells derived from the monocyte/macrophage lineage of hematopoietic stem cells. There are two key osteoclastogenic cytokines: macrophage colony-stimulating factor (M-CSF) and receptor for activation of nuclear factor κB (NF-κB) (RANK) ligand (RANKL). M-CSF causes the proliferation of early macrophage/osteoclast precursors, while RANKL acting via its receptor RANK on these precursors causes them to differentiate into mature osteoclasts. RANKL-RANK interaction activates a cascade of intracellular signaling pathways including nuclear factor (NF)-κB, PI-3 kinase/AKT, nuclear factor of activated T cells cytoplasmic 1 (NFATc1), and MAPKs. Among these pathways, NF-κB plays a pivotal role early in the initiation of osteoclastogenesis [3]. Recent studies have also identified NFATc1 as a key RANKL-mediated transcription factor. NFATc1 is capable of inducing transcription of diverse osteoclast-associated genes, such as those coding for tartrate-resistant acid phosphatase (TRAcP, Acp5), cathepsin K (Ctsk), and calcitonin receptor [4]. Therefore, inhibition of RANKL-mediated signalling molecules may contribute to the treatment and prevention of osteolytic bone diseases.

Berberine, an isoquinoline derivative alkaloid, is isolated from several natural herbs, such as *Cortex phellodendri* (Huangbai) and *Rhizomacoptidis* (Huanglian). Berberine has multiple pharmacological effects, including anti-bacterial [5], anti-tumour [6], and apoptosis-induction actions [7,8,9] but is poorly absorbed into the bloodstream when taken orally. Previous studies also suggested that berberine inhibits osteoclast formation and bone resorption [10], bone loss in ovariectomized (OVX) rats [11], and NF-κB and Akt pathways in osteoclasts [12].

In search for natural compounds that can inhibit osteolysis, we have conducted drug screening using combined osteoclastogenesis assays, and NF-κB and NFAT luciferase reporter gene assays. To this end, we have identified novel compounds that inhibit osteoclast formation and osteolysis [13,14]. In this study, we found that berberine sulfate, a derivative formula of unsulfated berberine that is easily absorbed, inhibits osteoclast differentiation. In addition, we determined the inhibitory effects of berberine sulfate on RANKL-induced osteoclast marker genes expression and NF-κB and NFAT pathways. Our results suggest a potential role for berberine sulfate in the treatment of osteoporosis.

## 2. Results

### 2.1. Berberine Sulfate Inhibits RANKL-Induced Osteoclastogenesis

**Figure 1:**
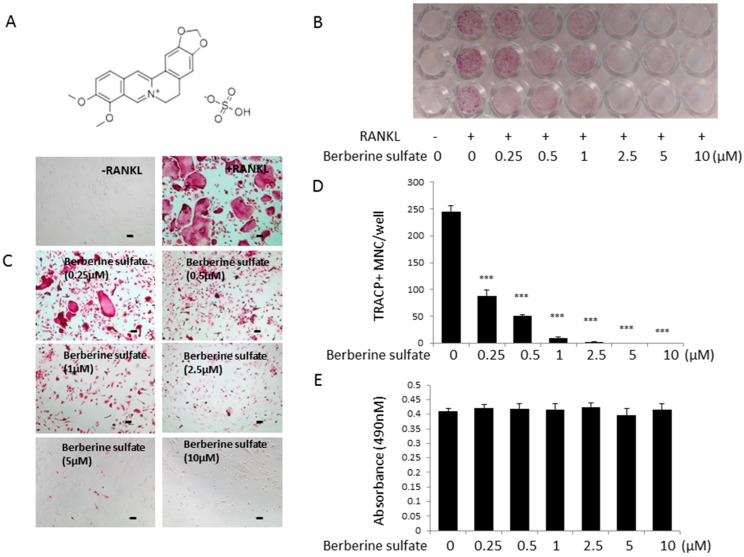
Berberine sulfate inhibits RANKL-induced osteoclastogenesis in BMM cells. (**A**) Chemical structure of berberine sulfate. The molecular weight of berberine sulfate is 433.43; (**B**) The 96 well-plate showing the effects of different concentration of berberine sulfate on the BMMs derived osteoclast-like cell formation. BMM cells (6 × 10^3^ cell/well) were cultured in the presence of M-CSF and GST-rRANKL (50 ng/mL) with or without different concentration of berberine sulfate for five days. Then, the cells were stained for TRAcP. “−“ means RANKL untreated; and“+” means RANKL treated; (**C**) Enlarged images of B (scale bar: 100 µm); (**D**) Osteoclast cell counts showing TRAcP-positive multinucleated cells. (*n* = 3); (**E**) Effect of berberine sulfate on the viability of BMM cells as measured by MTS assay. (*n* = 3). *** *p* < 0.001 (*versus* RANKL-treated control).

To examine the effect of berberine sulfate on the formation of osteoclasts, osteoclastogenesis assay was performed. Bone marrow macrophages (BMMs) were stimulated by RANKL for five days at the presence of varying concentrations of berberine sulfate, then fixed and stained for TRACP activity. Our results showed that the number of osteoclasts was significantly reduced in a dose-dependent manner, with an IC_50_ of 0.25 µM (Figure 1B–D). In addition, the size of osteoclasts was also decreased (Figure 1C). Cell proliferation assay (MTS) results showed that berberine sulfate has no effect on cell viability at doses up to 10 µM (Figure 1E). Thus, the inhibitory effect of berberine sulfate on osteoclast formation was not due to the toxicity of berberine sulfate.

### 2.2. Berberine Sulfate Suppresses RANKL-Induced Osteoclast Function

**Figure 2:**
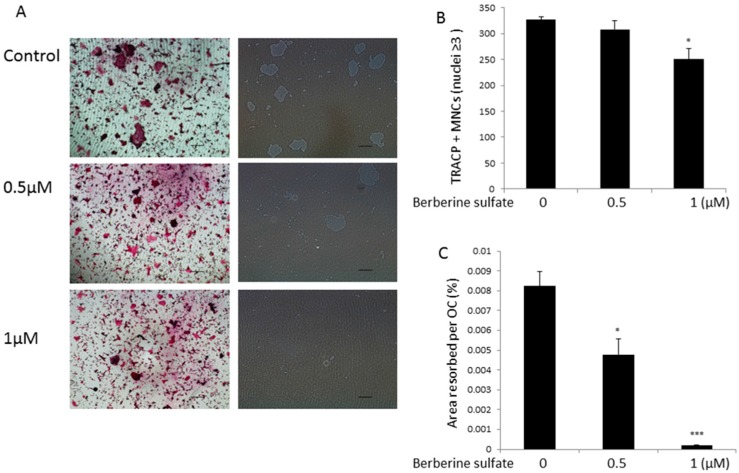
Berberine sulfate suppresses osteoclast function. (**A**) Representative images of osteoclastic resorption and TRAcP staining on hydroxyapatite coated surfaces (Scale bars, 500 µm); (**B**) The effect of berberine sulfate on the number of TRAcP positive multinucleated cells (Nuclei ≥ 3, counted as osteoclasts); (**C**) Percentage of the area of hydroxyapatite surface resorbed per osteoclast. * *p* < 0.05, *** *p* < 0.001 relative to untreated controls.

To study the effect of berberine sulfate on mature osteoclast resorptive function, mature osteoclasts were seeded on hydroxyapatite-coated plates and then treated with berberine sulfate for 48 h. Our results showed that the percentage of area resorbed per osteoclast was significantly reduced in the presence of berberine sulfate at the concentration of 0.5 µM and resorption was almost completely absent in the 1 µM group. A small reduction in osteoclast number was also observed at the dose of 1 µM (Figure 2). These results suggested that berberine sulfate suppresses mature osteoclast resorptive function.

### 2.3. Berberine Sulfate Suppresses RANKL-Induced Osteoclast-Associated Gene Expression

**Figure 3:**
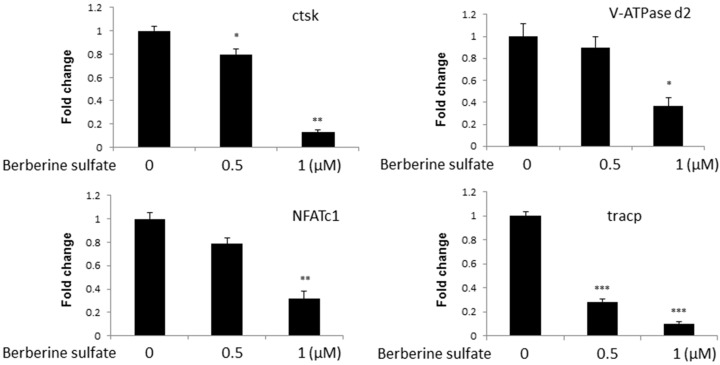
Effect of berberine sulfate on mRNA levels of osteoclast-associated genes. Real time-PCR analysis was performed to examine osteoclast-specific gene expression (Ctsk, V-ATPase d2, NFATc1, and TRAcP), and results were normalized to the expression of GAPDH. * *p* < 0.05, ** *p* < 0.01, *** *p* < 0.001 compared with untreated control.

To further investigate the effects of berberine sulfate on osteoclast formation, we examined the effect of berberine sulfate on osteoclast marker mRNA expression. Real time PCR analysis was performed on osteoclast culture. BMMs were stimulated with RANKL for five days in the presence of varying concentrations of berberine sulfate. Total RNA were extracted, and followed by real time PCR analysis. Our results showed that berberine sulfate reduces mRNA levels of cathepsin K (Ctsk), NFATc1, TRAcP and Vacuolar-type H^+^-ATPase V0 subunit D2 (V-ATPase d2), (Figure 3) consistent with the inhibitory effect of berberine sulfate on osteoclastogenesis.

### 2.4. Berberine Sulfate Inhibits RANKL-Induced NF-κB and NFAT Activity

**Figure 4:**
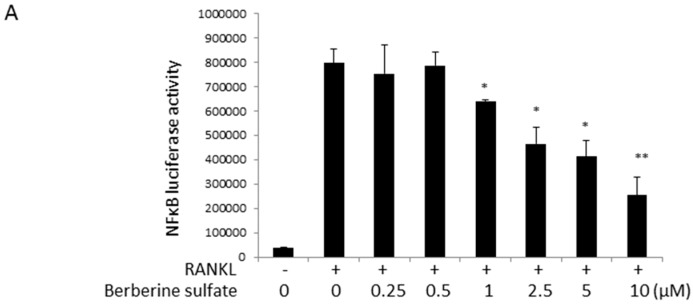

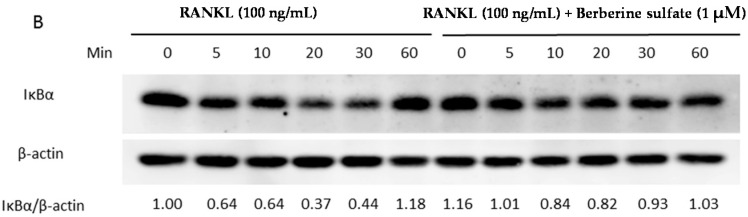
Berberine sulfate attenuated RANKL-stimulated NF-κB activity. (**A**) RAW264.7 cells stably transfected with an NF-κB transcriptional luciferase reporter construct, were pretreated with indicated concentration of berberine sulfate for 1 h and subsequently stimulated with RANKL (50 ng/mL) for an additional 6 h. The cells were harvested, and the luciferase activity was assayed. (*n* = 6). “−“ means RANKL untreated; “+” means RANKL treated. * *p* < 0.05, ** *p* < 0.01 *versus* untreated control; (**B**) BMM cells were pretreated with serum-free culture medium for 3 h, and berberine sulfate (1 µM) for 1 h and then were incubated with RANKL (100 ng/mL) for indicated times. The levels of IκB-α were evaluated by Western blot analysis

**Figure 5:**
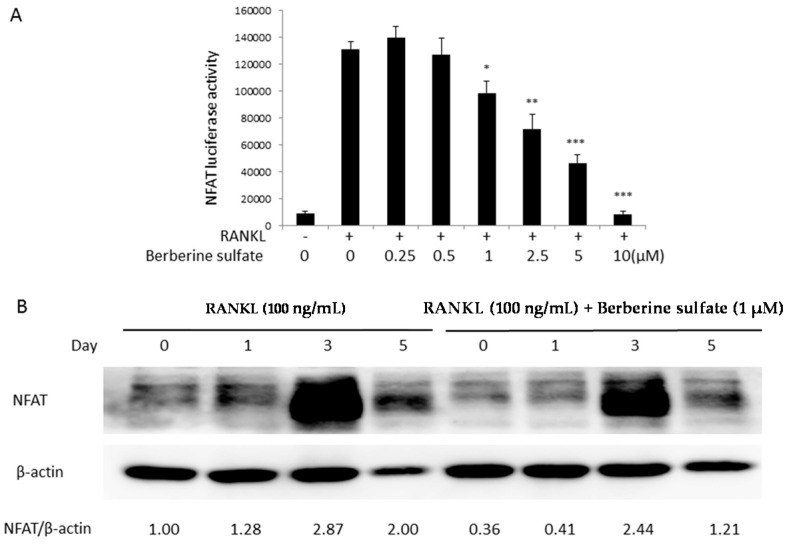
Berberine sulfate suppressed RANKL-induced NFAT activity. (**A**) RAW264.7 cells were stably transfected with an NFAT-dependent transcriptional luciferase reporter construct and then pretreated with indicated concentration of berberine sulfate for 1 h, prior to the addition of RANKL (50 ng/mL) for 24 h. The cells were harvested, and the luciferase activity was measured (*n* = 6). “−“ means RANKL untreated; “+” means RANKL treated. * *p* < 0.05, ** *p* < 0.01, *** *p* < 0.001 compared with untreated control; (**B**) BMM cells were pretreated with berberine sulfate for 1 h, followed by RANKL (100 ng/mL) stimulation for indicated times. The cell lysates were probed for NFATc1 protein levels using Western blot analysis.

To explore the molecular mechanism of action by which berberine sulfate inhibits osteoclast formation, the effects of berberine sulfate on RANKL-induced NF-κB and NFAT activities were tested using luciferase reporter gene assays. RAW264.7 cells stably transfected with a NF-κB luciferase reporter construct were seeded in 48 wells at 1.5 × 10^5^ cells/well and RAW264.7 cells stably transfected with an NFAT luciferase reporter construct were seeded at a density of 5 × 10^4^ cells/well and left overnight to attach. The next day, the cells were pre-treated with berberine sulfate for one hour, and then stimulated with RANKL for six hours for NF-κB luciferase assay and 24 h for NFAT luciferase assay, respectively. Our results showed that berberine sulfate significantly inhibited NF-κB (Figure 4A) and NFAT (Figure 5A) activities from the dose of 1 µM, with an IC50 of approximately 5 µM.

### 2.5. Berberine Sulfate Inhibits RANKL-Induced IKBα Protein Degradation and NFATc1 Protein Expression

To further study the role of berberine sulfate in RANKL-induced NF-κB and NFAT signal pathways, Western blot analysis was performed to test the effects of berberine sulfate on IκBα protein degradation and NFATc1 protein expression. For detecting IκBα protein degradation, BMMs were stimulated by RANKL at 5, 10, 20, 30 and 60 min in the presence or absence of berberine sulfate. Our results showed that IκBα degradation was inhibited by berberine sulfate at the concentration of 1 μM (Figure 4B). The inhibition was especially clear at 20 and 30 min of RANKL stimulation. For detecting NFATc1 protein expression, BMMs were stimulated with RANKL at Day 1, 3 and 5 in the presence or absence of berberine sulfate. The expression of NFATc1 was also reduced by berberine sulfate at the concentration of 1 μM (Figure 5B). These results further attested the inhibitory effect of berberine sulfate on RANKL-induced NF-κB and NFAT signal pathways.

## 3. Discussion

Postmenopausal osteoporosis is a disease featured by an imbalance between the activities of osteoclasts and osteoblasts, with excessive osteoclastic bone resorption predominating over osteoblast bone formation. Therefore, osteoclasts are prime targets of commonly used anti-catabolic drugs such as bisphosphonates (BPs), and Denosumab, a fully human anti-RANKL IgG2 monoclonal antibody. In this study, through screening of natural compounds that exhibit inhibitory effects on osteoclastogenesis and RANKL signalling, we found that berberine sulfate is capable of inhibiting osteoclast formation, osteoclast marker genes expression, and RANKL-induced NF-κB and NFAT activities, suggesting that berberine sulfate is a potential anti-catabolic agent for the treatment of osteoporosis.

Berberine was previously reported to have a wide range of pharmacological effects [15]. However, absorption of berberine via the intestinal wall is very poor [16]. Indeed, after oral administration, berberine is typically undetectable in blood [17]. The metabolites of berberine after absorption when detectable are mainly sulfate or glucuronide conjugates [18]. For these reasons, berberine sulfate is a commonly used salt form of berberine as this form is both stable and clearly detected in the blood after oral administration. While berberine itself has been examined for its effects on osteoclast formation, its sulfated derivative has not. In this study, we found that berberine sulfate strongly inhibits osteoclastogenesis with an IC_50_ of approximately 0.25 µM.

RANKL, a member of the tumour necrosis factor (TNF) super family, was identified as a critical cytokine for osteoclastogenesis [19]. The binding of RANKL and RANK activates several downstream molecules which are important for regulating osteoclast differentiation, function and survival, including NF-κB, AP-1, TRAFs, NFATc1 and ionized calcium [3,20]. NF-κB pathway is the most important among RANKL-induced early signaling pathways, as without this signal no osteoclast formation can occur. RANKL binding causes the binding of its receptor RANK to TRAF6 which interacts with TAB2, forming a complex to activate the downstream TAKl, inducing the phosphorylation of IKKα and β. The activated IKKα and β induce the phosphorylation of IκB [21,22]. The phosphorylated IκB is then degraded via proteasome pathway, allowing the release of NF-κB to the nucleus [23,24,25]. In our study, we found that NF-κB activity and IκBα protein degradation were inhibited by berberine sulfate. However, the phosphorylation of ERK was not inhibited by berberine sulfate (Figure S1). These results are consistent with a previous study, which suggested that berberine prevented the IκBα degradation but not the phosphorylation of ERK and P38 [12].

NFATc1, a RANKL-induced transcription factor that is dependent upon prior NF-κB activation, plays a key regulatory role in osteoclast differentiation and indeed can drive osteoclast formation when it is overexpressed in osteoclastic progenitor cells [4]. Its stability and nuclear translocation is also dependent on Ca^2+^ dependent calcineurin activity [4], making it essential to use activity assays (such as the luciferase based system employed here) to investigate the effects of an anti-osteoclastic factor such as berberine sulfate. Together with NF-κB, AP-1 and other transcription factors NFATc1 elicits the expression of osteoclast-associated genes such as TRAcP (Acp5), MMP9, calcitonin receptor, Ctsk, V-ATPase d2, c-Src and other genes that are required for osteoclast function. In this study, we found that berberine sulfate suppressed RANKL-induced NFATc1 transcriptional activity and NFATc1 protein levels. It is not clear whether berberine sulfate acts directly on NFATc1 expression or stability, since it is possible that its primary action is to suppress NF-κB and only indirectly NFATc1. However, consistent with reduced NFATc1 protein levels, berberine sulfate was observed to reduce mRNA expression of osteoclast associated proteins Ctsk, V-ATPase d2, and TRAcP (Acp5), downstream targeted genes of NFATc1 activity, which is consistent with the inhibitory effects of berberine sulfate on NFATc1 transcriptional activity. However, the exact molecular targets of berberine in osteoclasts are not currently known. Further work is required to determine if berberine sulfate is internalized to inactivate RANKL-dependent signaling pathways intracellularly, or whether it interferes with cell surface interactions such as RANKL binding to RANK, or binding to other specific transmembrane receptors.

## 4. Materials and Methods

### 4.1. Media and Reagents

Berberine sulfate (Purity > 98%) was purchased from Mansite (Chengdu, China), and dissolved in Dimethyl sulfoxide (DMSO). α Modification of Minimal Essential Medium (α-MEM) and fetal bovine serum (FBS) was purchased from TRACE (Sydney, Australia). Recombinant GST-rRANKL protein was expressed and purified as previously described [26] and recombinant macrophage colony stimulating factor (M-CSF) was obtained from R&D Systems (Minnneapolis, MN, USA). MTS reagent and luciferase analysis reagents were obtained from Promega (Sydney, Australia). Antibodies against NFATc1, IκBα, ERK, phosphorylated ERK, and β-actin were obtained from Santa Cruz Biotechnology (Dallas, CA, USA). Tartrate resistant acid phosphatase (TRAcP) enzymatic activity was detected using the Leukocyte acid phosphatase staining kit (Sigma, St. Louis, MO, USA).

### 4.2. Cell Culture

RAW264.7 cells were obtained from the American Type Culture Collection (Rockville, MD, USA). RAW264.7 cells and freshly isolated bone marrow macrophages (BMM) from C57BL/6 mice were grown in α-MEM supplemented with 10% heat inactivated FBS, 2 mM l-glutamine and 100 U/mL penicillin/streptomycin (complete α-MEM). Primary BMM were grown in complete α-MEM with the addition of macrophage-colony stimulating factor (M-CSF). All cell cultures were maintained in 5% CO_2_ at 37 °C.

### 4.3. In Vitro Osteoclastogenesis Assay

BMM cells (at a density of 6 × 10^3^ cell/well in 96-well plate) were seeded and cultured in the presence of complete α-MEM medium containing M-CSF and GST-rRANKL (50 ng/mL) with or without different concentration of berberine sulfate. The medium as above described was changed every 2 days. After 5 days, cultured cells were fixed for with 4% paraformaldehyde for 10 min at room temperature. Fixed cells were then histochemically stained as per manufacturer’s instructions using a TRAcP staining kit (Sigma, St. Louis, MO, USA). The osteoclast-like cells (OCL) were quantified as the total number of TRACP-positive multinucleated cells (more than 3 nuclei).

### 4.4. Cytotoxicity Assays

BMMs were plated onto 96-well plate (6 × 10^3^ cell/well) overnight, and then treated with complete α-MEM medium with M-CSF (25 ng/mL) and different concentration of berberine sulfate for 48 h. Next, 20 µL of MTS ((3-(4,5-dimethylthiazol-2-yl)-5-(3-carboxymethoxyphenyl)-2-(4-sulfophenyl)-2*H*-tetrazolium), was added and incubated for 2 h. Cell viability assay was measured by optical density at 490 nm using a microplate reader (ThermoFisher, Waltham, MA, USA).

### 4.5. Hydroxyapatite Resorption Assay

For the experiment, 1 × 10^5^ cells/well BMMs were seeded onto 6-well collagen-coated culture plates overnight at 37 °C. Then, the cells were stimulated with 50 ng/mL GST-rRANKL and M-CSF until osteoclasts were formed. The cells were gently harvested using cell dissociation buffer, and equal numbers of multinucleated cells were seeded on hydroxyapatite-coated plates (Corning, New York, NY, USA). The cells were then treated with different concentrations of berberine sulfate in the presence of 50 ng/mL GST-rRANKL and M-CSF. After 48 h, half of the wells were fixed and stained for TRAcP activity for osteoclast counting and the remainder of the wells were bleached and dried for hydroxyapatite resorption visualisation using a Nikon microscope (Nikon Corporation, Tokyo, Japan) and analysed using Image J software ( National Institutes of Health, Bethesda, MD, USA).

### 4.6. RNA Isolation and Analysis

BMMs were cultured with berberine sulfate at various concentration in the presence of M-CSF (25 ng/mL) and GST-rRANKL (50 ng/mL) for 5 days on 6 well plate (1 × 10^5^ cell/well). Then, total RNA from cells were isolated using Trizol regent (Life Technologies, Mulgrave, Australia). cDNA was synthesized from 1 μg of RNA using reverse transcriptase with oligo-dT primer. All PCR reactions used specific primers following the mouse sequences: Cathepsin K (Ctsk) (forward: 5′-GGG AGA AAA ACC TGA AGC-3′; reverse: 5′-ATT CTG GGG ACT CAG AGC-3′), TRAcP (Acp5) (forward: 5′-TGT GGC CAT CTT TAT GCT-3′; reverse: 5′-GTC ATT TCT TTG GGG CTT-3′), NFATc1 (forward: 5′-CAA CGC CCT GAC CAC CGA TAG-3′; reverse: 5′-GGC TGC CTT CCG TCT CAT AGT-3′), V-ATPase-d2 (forward: 5′-GTG AGA CCT TGG AAG ACC TGA A-3′; reverse: 5′-GAG AAA TGT GCT CAG GGG CT-3′), GAPDH (forward: 5′-ACC ACA GTC CAT GCC ATC AC-3′; reverse: 5′-TCC ACC ACC CTG TTG CTG TA-3′). qPCR reactions were performed on a ViiA™ 7 Real-time PCR machine (Applied Biosystems, Paisley, UK). The comparative 2^−ΔΔ*C*t^ method was used to calculate the relative expression of each target gene. The mean *C*_t_ value of target genes in the experimental group was normalized to the *C*_t_ value of GAPDH to give a Δ*C*_t_ value, which was further normalized to control samples to obtain ΔΔ*C*_t_. Three independent cultures were carried out, and all experiments were performed in triplicate.

### 4.7. NF-κB and NFAT Luciferase Reporter Gene Assays

NF-κB and NFAT activation were measured by luciferase reporter gene assays. Briefly, RAW264.7 cells stably transfected with an NF-κB luciferase construct (3κB-Luc-SV40) [27] and with an NFATc1 luciferase reporter construct [28]; respectively. The transfected cells were plated in 48-well plates (1.5 × 10^5^ and 5 × 10^4^ cells/well; respectively). Then, cells were pre-treated with different concentration of berberine sulfate for 1 h, followed by GST-rRANKL (50 ng/mL) stimulation for 6 h (NF-κB luciferase report gene assay) or 24 h (NFAT luciferase reporter gene assay). Analysis of luciferase activity was performed in accordance with the manufacturer’s instructions (Promega, Sydney, Australia).

### 4.8. Western Blot Assays

Protein was extracted from cells in culture by scraping the cells into radioimmunoprecipitation assay (RIPA) buffer. Samples were resolved using sodium dodecyl sulfate polyacrylamide gel electrophoresis (SDS-PAGE) and transferred to nitrocellulose membranes; these were then blocked with 5% skim milk for 1 h at room temperature and probed with primary antibodies overnight at 4 °C. After three washes, membranes were incubated with secondary antibodies conjugated with horseradish peroxidase (HRP) for 1 h. Protein bands were then visualized using the enhanced chemiluminescence (ECL) system (Amersham Pharmacia Biotech, Sydney, Australia).

### 4.9. Statistical Analysis

All data were shown as the mean ± SEM with at least 3 independent experiments. Statistical analysis used the student’s *t* test to assess statistical differences, *p* values less than 0.05 were considered to be significant.

## 5. Conclusions

We have identified that berberine sulfate, a commonly used salt form of berberine, is capable of inhibiting osteoclast formation and the expression of osteoclast marker genes through the suppression of RANKL-induced NF-κB and NFAT activation. Our study suggests that berberine sulfate is a potential therapeutic agent for the treatment of osteoporosis.

## Supplementary Materials

Supplementary materials can be found at http://www.mdpi.com/1422-0067/16/11/25998/s1.
