# Supplementary material for: Berberine Sulfate Attenuates Osteoclast Differentiation through RANKL Induced NF-κB and NFAT Pathways
Source: Int J Mol Sci. 2015 Nov 13;16(11):27087–96. doi: 10.3390/ijms161125998 (PMC4661856; doi:10.3390/ijms161125998)
Supplement: Supplementary file 1 [file ijms-16-25998-s001.pdf]

Supplementary Materials: Berberine Sulfate Attenuates Osteoclast Differentiation through RANKL Induced NF-κB and NFAT Pathways

Lin Zhou, Fangming Song, Qian Liu, Mingli Yang, Jinmin Zhao, Renxiang Tan, Jun Xu, Ge Zhang, Julian M. W. Quinn, Jennifer Tickner and Jiake Xu

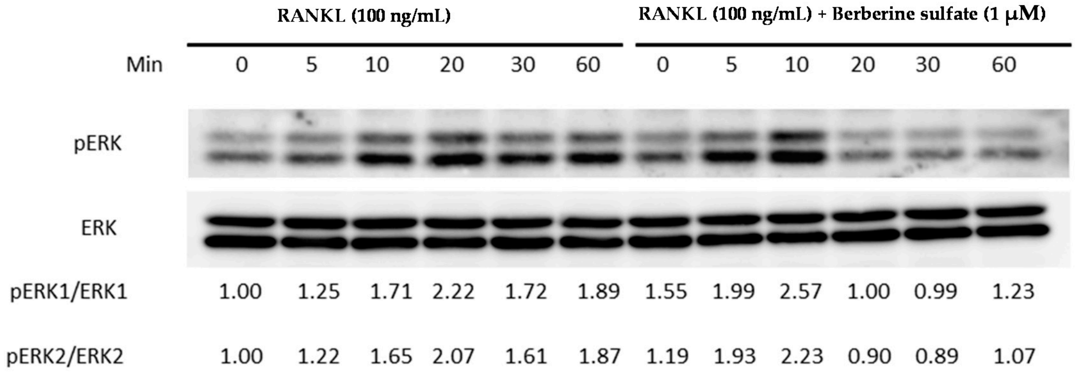

Figure S1. Berberine sulfate didn't inhibit the phosphorylation of ERK. BMM cells were pretreated with serum-free culture medium for 3 h, then berberine sulfate (1 μM) for 1 h, and subsequently incubated with RANKL (100 ng/mL) for indicated times. The levels of pERK and ERK were evaluated by Western blot analysis.
